# Effects of intradialytic exercise on cardiopulmonary capacity in chronic kidney disease: systematic review and meta-analysis of randomized clinical trials

**DOI:** 10.1038/s41598-019-54953-x

**Published:** 2019-12-05

**Authors:** Francini Porcher Andrade, Patrícia de Souza Rezende, Tatiane de Souza Ferreira, Gabrielle Costa Borba, Alice Mânica Müller, Paula Maria Eidt Rovedder

**Affiliations:** 1 Programa de Pós Graduação em Ciências Pneumológicas at Universidade Federal do Rio Grande do Sul (UFRGS), Porto Alegre, postcode 90040-060 Brazil; 2 Physiotherapy Course, Universidade Federal do Rio Grande do Sul (UFRGS), Porto Alegre, postcode 90040-060 Brazil; 3 Hospital de Clínicas de Porto Alegre, Porto Alegre, postcode 90035-903 Brazil

**Keywords:** Rehabilitation, Haemodialysis, Fatigue

## Abstract

Patients with chronic kidney disease show poorer functional and cardiorespiratory capacity than healthy individuals, and these impairments result in sedentarism. The aim of this study was to conduct a systematic review and meta-analysis of randomized clinical trials on the effects of different intradialytic exercise protocols on cardiopulmonary capacity in chronic kidney disease patients. The primary outcome was peak oxygen consumption (VO_2peak_) and the secondary outcomes were exercise duration and ventilation in the cardiopulmonary test. The quality of the evidence was evaluated using the GRADE guidelines. Seven studies with a total of 124 participants met the inclusion criteria. Compared to the non-exercise group, the exercise group improved in mean VO_2peak_ (MD 4.06 [IC 0.81; 7.31]). In a separate analysis according to exercise modality, aerobic exercise plus strength training performed better than aerobic exercise alone (MD 5.28 [IC 3.90; 6.66]). In the exercise group, both exercise tolerance values (MD 3.10 [IC 1.70; 4.51]) and ventilation values in the cardiopulmonary test were better than those of the control group (MD 13.10 [IC 7.12; 19.09]). Thus, intradialytic exercise protocols can improve cardiopulmonary function, exercise tolerance and ventilatory efficiency in chronic kidney disease patients.

## Introduction

Patients with chronic kidney disease (CKD) have a slow, progressive and irreversible loss of renal function, causing metabolic and hydroelectrolytic imbalances. The prevalence of CKD has been increasing in recent years and, in most cases, its diagnosis is late, when renal replacement therapy is necessary through peritoneal dialysis, hemodialysis or renal transplantation^1^.

CKD patients who undergo hemodialysis have poorer functional capacity, which is related to deconditioning and low tolerance for physical activity^2^. Sedentary behavior is either the cause or consequence of disease progression, and poor functional capacity is associated with increased mortality^3^.

Multiple systems, including cardiovascular and respiratory function, are impaired in CKD patients on dialysis, which is induced by complications such as accumulated uremic toxins and other impurities, volume overload from fluid retention, anemia from lack of erythropoietin production and hyperparathyroidism. This is due to both hemodialysis treatment (e.g. immobility and post-dialysis fatigue) and the disease itself (uremic neuro and myopathy, anemia, cardiovascular abnormalities and electrolyte imbalance)^4–6^.

Cardiovascular disease it is the main cause of morbidity and mortality in CKD, with nearly double the incidence of the general population^7^. Moreover, CKD patients with cardiovascular comorbidities have shown progressive worsening in functional capacity^8^. Respiratory complications are also common, such as interstitial pulmonary edema and restrictive spirometric patterns^5^.

The cardiopulmonary exercise test (CPET) can be used to objectively determine functional capacity, which involves the pulmonary and cardiovascular systems. The peak oxygen consumption (VO_2peak_) value obtained in the CPET defines a person’s functional aerobic capacity and has become the gold standard for cardiopulmonary fitness^9^. Studies show that VO_2peak_ values greater than 17.5 ml/min/Kg are predictors of survival in CKD patients, indicating that it is essential to evaluate both functional capacity and its evolution with the CPET in these patients^10^.

The aim of this study was to conduct a systematic review and meta-analysis of randomized clinical trials (RCTs) on the effects of different intradialytic exercise protocols on cardiopulmonary capacity in CKD patients. Our meta-analysis expands the results by assessing the patients’ cardiopulmonary function, which often is ignored in this population.

## Methods

This systematic review and meta-analysis of RCTs is registered with the International Prospective Register of Ongoing Systematic Reviews (number CRD42019119212) and followed the PRISMA Statement and the Cochrane Collaboration recommendations^11^.

### Eligibility criteria

The review included RCTs that involved chronic renal failure patients who underwent intradialytic exercise protocols, evaluated VO_2peak_ (mLkg/min or liters) through cardiopulmonary testing, and featured a control group.

The exclusion criteria were pediatric populations, modified drug regimens, or not using a maximal exercise test to obtain VO_2peak_.

The primary outcome measure was VO_2peak_ in mL/kg/min; the secondary outcome measures were exercise duration and ventilation in the cardiopulmonary test.

### Search strategy

The studies were found through a systematic search of MEDLINE (accessed through PubMed), the Cochrane Central Register of Controlled Trials, and EMBASE, in addition to a manual search of the references in published studies on the subject. No publication date or language restrictions were set. The PubMed search included clinical trials, controlled clinical trials and randomized controlled trials, the Cochrane search included trials, and the EMBASE search included randomized controlled trials. Studies were eligible if they were published from the beginning of the databases until September 2018 and involved the following descriptors or synonyms: “Renal Insufficiency Chronic”, “Exercises”, “Physical Activity”, “Cardiopulmonary Exercise Tests”. The search strategy is shown in the Supplementary Information.

### Study selection and data extraction

The titles and abstracts of all articles identified in the search strategy were independently evaluated by two investigators (F.P.A and T.S.F.), strictly adhering to the inclusion and exclusion criteria. For articles that did not provide enough information in the titles and abstracts, a full-text assessment was performed by the same investigators. Disagreements over inclusion were resolved by consensus among the investigators and an independent third reviewer (A.M.M).

Two investigators (F.P.A. and T.S.F.) performed the data extraction independently using standardized forms. The primary extracted endpoint was peak VO_2_ (mL/kg/min and liters) and the secondary outcomes were cardiopulmonary test duration and peak ventilation in the cardiopulmonary test. The structured data collection form included the following study characteristics: country in which the study was conducted, date of enrolment, study design, study setting, and patient population features. The extracted numerical data included: number of patients in each study, number of patients in each group, VO_2_ delta value (mL/kg/min and liters), cardiopulmonary test duration delta value, delta value of peak ventilation in the cardiopulmonary test, and the exercise modality, frequency and duration.

### Risk of bias assessment

The studies’ methodological quality was evaluated independently and descriptively by the same two reviewers based on Cochrane Collaboration recommendations^11^. The following items were evaluated: random sequence generation, allocation concealment, patient blinding, blinding of therapists and outcome assessors, intention-to-treat analysis, and description of losses and exclusions. If any of these items were not clearly described, they were considered not informed. Intention-to-treat analysis was defined as confirmation in the study assessment that the number of randomized participants and the number of analyzed participants were identical. Quality assessment was performed independently by two reviewers (F.P.A and T.S.F). The dates are shown in Table 1 - Supplementary Information.

### Summary of evidence: GRADE criteria

The quality of the evidence was evaluated according to Grading of Recommendations Assessment, Development and Evaluation (GRADE) criteria and the Cochrane Handbook for Systematic Reviews of Interventions. For each specific outcome, the quality of evidence was based on five factors: (1) risk of bias; (2) inconsistency; (3) indirectness; (4) imprecision; and (5) publication bias. The GRADE approach resulted in four levels of quality of evidence: high, moderate, low and very low, and was performed at https://gdt.gradepro.org/app/. The data are shown in Table 2 - Supplementary Information.

### Data analysis

Binary outcomes for each trial were expressed as odds ratios and 95% confidence intervals. The data from all trials were pooled as appropriate using a fixed effect model and a random effects model. Meta-analysis was performed for all outcomes in R version 3.5.0. When the standard deviation of the mean was not available, the standard error of the mean was used for the meta-analysis. The studies compared exercise training groups and control groups that did not exercise. The inconsistency test (I^2^) was used to assess heterogeneity among the studies; values ≥50% indicated high heterogeneity.

## Results

### Study selection

**Figure 1:**
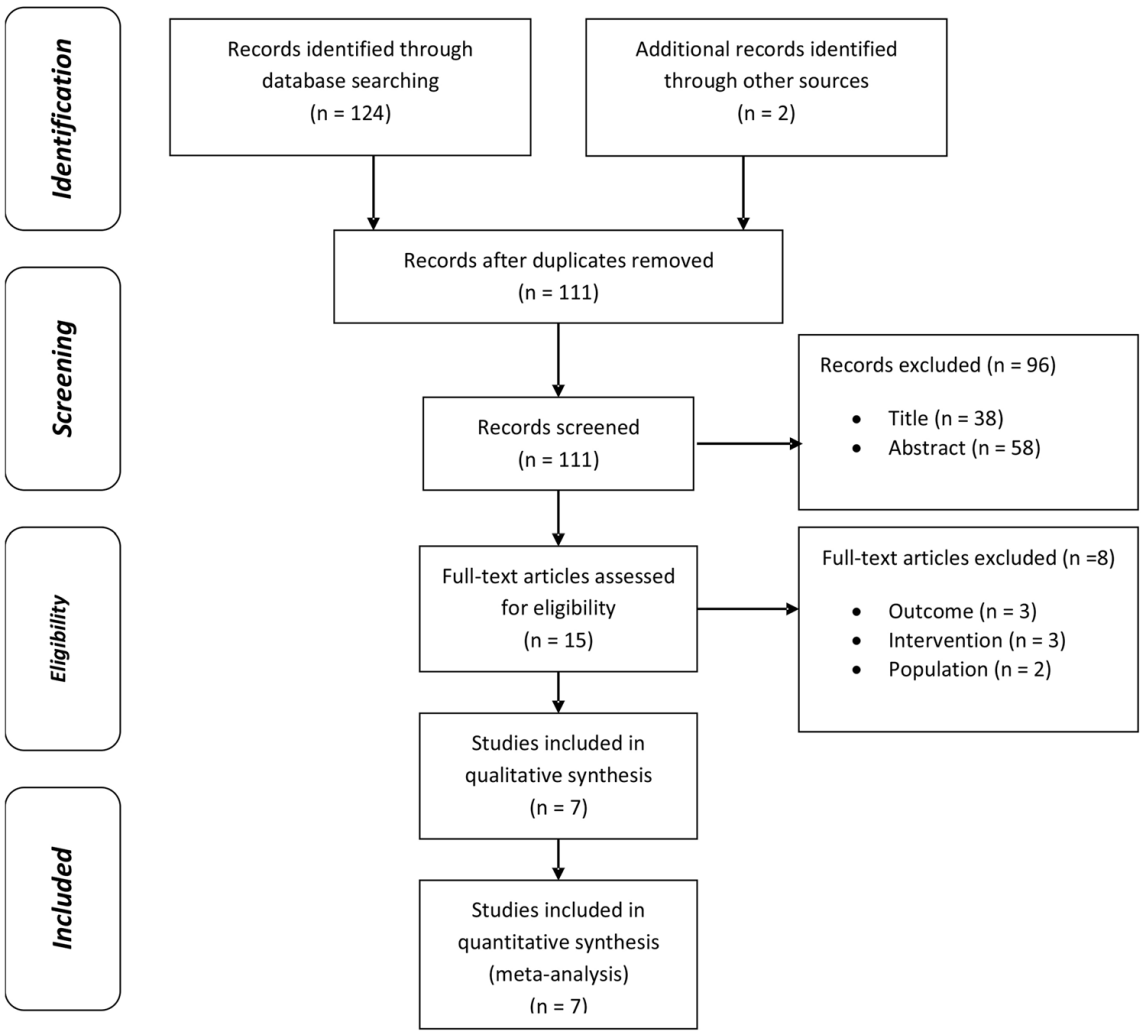
Study selection flowchart.

Figure 1 presents a flowchart of the included studies. A total of 126 studies were initially selected through the PubMed, EMBASE, and Cochrane database searches and the manual search. After removing fifteen duplicate publications, 111 studies remained, of which 39 were excluded after assessing the title and 58 after assessing the abstract. Fifteen studies remained for full text review, of which seven were included and meta-analyzed.

Seven studies were included in the meta-analysis. Figure 1 shows the study selection flowchart.

To define the intensity of the exercise protocol, the majority of the studies used the Borg Scale, which is the most appropriate instrument for CKD patients. Only two studies used the cardiopulmonary exercise test to determine the training intensity, which should be considered unreliable for this population due to the fact that beta-blocker drugs, which are widely used by these patients, interfere in the maximum heart rate. These authors did not explain how they obtained a workload of 55–60% peak power^12,13^.

All studies began the exercise protocol within two hours of hemodialysis to avoid cardiovascular instability.

The seven studies, published between 2008 and 2015, involved 243 patients from Brazil, France and Greece: 125 in the exercise group and 118 in the control group. The studies included a majority of males: 79 men in the exercise group and 74 in the control group, compared to 46 women in the exercise group and 44 in the control group. Table 1 shows the details of the included studies and their samples.

**Table 1:** Detailed characteristics of the included studies.

Author	Country	Follow-up	Group	N	Exercise modality	Frequency	Intensity	Exercise time	Time on dialysis	Age	Sex (male)
Groussard *et al*.	France	3 months	E	8	Intradialytic cycle training	3 times/week for 3 months	55–60% of peak power output	30 min	36.6 ± 8.2*	68.4 ± 3.7	5
(2015)			C	10	Regular dialysis treatment				41.2 ± 8.1*	66.5 ± 4.6	7
Reboredo *et al*.	Brazil	12 weeks	E	12	Intradialytic cycle training	3 times/week for 12 weeks	Modified Borg scale (between 4–6)	35 min	3.3 ± 3.4^#^	50.7 ± 10.7	5
(2011)			C	12	Regular dialysis treatment				4.8 ± 4.4	42.2 ± 13	5
Kouidi *et al*.	Greece	1 Year	E	24	Cycling training and strength training	3 times/week for 1 year	Borg scale (between 11–13)	60–90 min	6.1 ± 4.6^#^	46.3 ± 11.2	14
(2010)			C	20	Regular dialysis treatment				6.3 ± 4.9^#^	45.8 ± 10.9	12
Kouidi *et al*.	Greece	10 months	E	30	Cycling training and strength training	3 times/week for 10 months	Borg scale (between 11–13)	60–90 min	6.3 ± 3.7^#^	54.6 ± 8.9 5	18
(2009)			C	29	Regular dialysis treatment				6.2 ± 3.9^#^	53.2 ± 6.1	16
Ouzouni *et al*.	Greece	10 months	E	19	Cycling training, strength training and flexibility	3 times/week for 10 months	Borg scale (between 13–14)	60–90 min	7.7 ± 7.0#	47.4 ± 15.7	14
(2008)			C	14	Regular dialysis treatment				8.6 ± 6.0^#^	50,5 ± 11,7	13
Petraki *et al*.	Greece	7 months	E	22	Cycling training, strength training and flexibility	3 times/week for 10 months	13 at Borg scale	90 min	76.32 ± 7.0*	50.05 ± 3.2	15
(2008)			C	21	Regular dialysis treatment				10.5 ± 15.1*	50.52 ± 14.4	17
Konstantinidou *et al*.	Greece	6 months	E	10	Cycling training, strength training and flexibility	3 times/week for 6 months	Approximately 70% of HRmax	60 min	77 ± 66*	48.3 ± 12.1	8
(2002)			C	12	Regular dialysis treatment				79 ± 86*	50,2 ± 7,9	4

Legend: E: experimental group; C: control group, *time on dialysis in month; ^#^time on dialysis in year.

**Figure 2:**
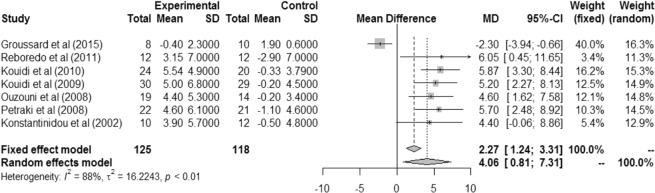
Difference in VO_2peak_ mL/kg/min between pre- and post-intervention.

All analyses were performed using the delta values. The heterogeneity of the VO_2peak_ delta values (mL/kg/min) was significant (I² = 88%, r² = 16.2243, p < 0.01). However, despite the high heterogeneity, there were significant differences between the exercise and control groups in the fixed effect model (MD 2.27 [IC 1.24; 3.31]) and in the random effects model (MD 4.06 [IC 0.81; 7.31]), demonstrating that exercise during hemodialysis can benefit physical functioning (Fig. 2).

**Figure 3:**
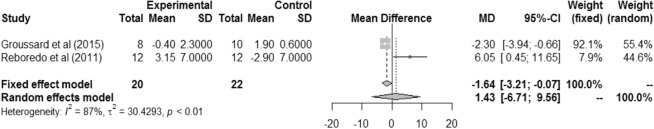
Difference in VO2peak mL/kg/min between intervention and control groups in patients who performed aerobic exercise alone.

Separate analysis according to exercise modality was also performed (aerobic exercise only or aerobic exercise plus strength training). There was a significant difference in VO_2peak_ mL/kg/min value for aerobic exercise only in the fixed effect model (MD −1.64 [IC −3.21; −0.07]), with high and significant heterogeneity (I² = 87%, r² = 30.42, p < 0.01) (Fig. 3).

**Figure 4:**
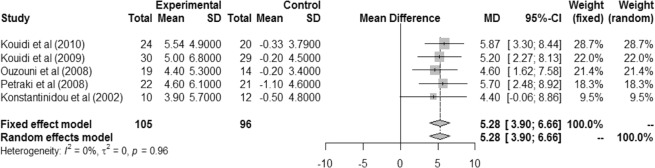
Difference in VO2peak mL/kg/min between intervention and control groups in patients who performed aerobic exercise and strength training.

Benefits to cardiopulmonary capacity were also found in studies that combined aerobic exercise and strength training, and these results were significant in both models (fixed and random effects), with similar values in fixed effect and random effects models (MD 5.28 [IC 3.90; 6.66]). The heterogeneity was not significant (I² = 0%, r² = 0, p = 0.96) (Fig. 4).

**Figure 5:**
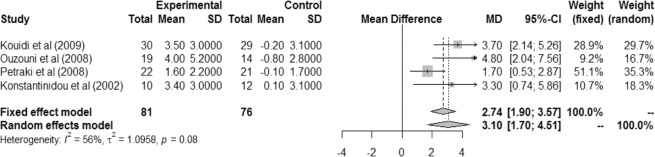
Difference in cardiopulmonary test duration (in minutes) between intervention and control groups.

There was a significant difference in cardiopulmonary test duration (in minutes) between the exercise and control groups in the fixed effect model (MD 2.74 [IC 1.90; 3.57]) and the random effects model (MD 3.10 [IC 1.70; 4.51]), with non-significant heterogeneity (I² = 56%, r² = 1.0958, p = 0.08). These results demonstrate that an exercise protocol during hemodialysis leads to greater exercise tolerance (Fig. 5).

**Figure 6:**
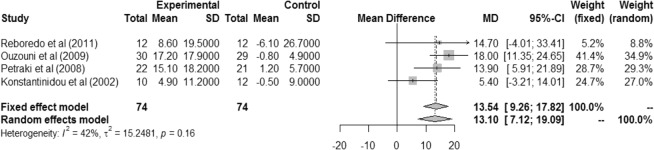
Difference in ventilation between pre- and post-intervention or between intervention and control groups.

There was a significant difference in ventilation (in liters) during the cardiopulmonary test between the exercise and control groups in the fixed effect model (MD 13.54 [IC 9.26; 17.82]) and the random effects model (MD 13.10 [IC 7.12; 19.09]), with non-significant heterogeneity (I² = 42%, r² = 15.2481, p = 0.16). This result highlights the importance of intradialytic exercise for pulmonary ventilation (Fig. 6).

Based on the GRADE criteria, the quality of the included studies ranged from very low to low. Of the included studies, 100% presented adequate sequence generation, 14% reported allocation concealment, 14% reported blinded assessment of outcomes and 100% described losses to follow-up and exclusions. The quality and the risk of bias assessments are provided in the Supplementary Information.

## Discussion

This systematic review with meta-analysis of RCTs indicates that interventions combining intradialytic aerobic exercise and strength training effectively increase cardiopulmonary capacity and exercise tolerance. There were also significant results for ventilation during CPET (in liters), demonstrating that exercise during hemodialysis can benefit the physical functioning of these patients.

The effects of intradialytic exercise were studied because this modality has better adherence among CKD patients than protocols performed outside hemodialysis centers^14^. Our meta-analysis demonstrates the benefits of intradialytic exercise and expands its results with a more thorough analysis of cardiopulmonary function in CKD patients^15–17^.

VO_2peak_ is the main evaluation parameter of cardiopulmonary function. It is considered the gold standard for evaluating cardiopulmonary fitness and directly assesses aerobic capacity. Using submaximal exercise protocols to assess cardiopulmonary function is unreliable, since they are limited by physiological mechanisms and methodological inaccuracies. The main determinants of VO_2peak_ are genetic factors, age, sex, body composition and greater activation of the neuromuscular mechanism, which can be improved through physical training^9^. Moreover, VO_2peak_ is inversely linked with cardiovascular risk and all-cause mortality^18^.

The results of this review showed that intradialytic exercise three times a week for at least three months led to a significant increase in VO_2peak_, despite the high and significant heterogeneity of the included studies.

The low cardiopulmonary capacity of hemodialysis patients has a number of causes, including anemia, muscular atrophy, cardiac dysfunction due to hypervolemia, metabolic disorders, reduced cardiac response to exercise and physical deconditioning^19^.

The effects of exercise on aerobic capacity in end-stage renal disease are related to important cardiovascular outcomes, as well as to improved cardiac performance and output. Thus, the maximal cardiopulmonary stress test could be a useful approach for risk stratification in CKD patients, providing prognostic information and predicting survival^10,18^.

In the stratified analysis according to training modality, intradialytic aerobic exercise alone did not significantly alter VO_2peak_ values in the random effects model. This corroborates the results of Groussard *et al*. (2015), whose aerobic exercise group showed improvement only in the submaximal exercise test^12^. This finding shows that aerobic exercise alone may only benefit activities of daily living in these patients and not cardiopulmonary fitness.

However, Sheng *et al*. (2014) found contrary results, i.e. that aerobic exercise alone can improve VO_2peak_^15^. Their review included studies by Van Vilsteren *et al*. (2005), Painter *et al*. (2002) and Koufaki *et al*. (2002), which were excluded from this meta-analysis due to methodological biases, such as obtaining VO_2peak_ with a submaximal test^20^ and altered drug regimens^21^, as well as for allocating patients who underwent peritoneal dialysis and hemodialysis in the same group^22^. Moreover, in Vilsteren *et al*. (2005) only aerobic training occurred during dialysis; strength training was performed during the pre-hemodialysis period^20^.

The meta-analysis of studies that combined intradialytic aerobic exercise and strength training revealed more relevant and favorable results regarding VO_2peak_ improvement. The results were significant in both models (fixed and random effects). Gomes Neto *et al*. (2018), whose meta-analysis also included a stratified analysis according to training modality in a random effects model, found similar results to the present study, although they also included studies by Painter *et al*. (2002) and Van Vilsteren *et al*. (2005)^17,20,21^.

Strength training helps improve the oxidative capacity of muscle due to increased oxygen use, which contributes to less muscle fatigue and, consequently, greater exercise tolerance^9,10,23^. Moreover, any exercise modality can improve nutrition and blood flow to the muscles, as well as increase circulation from small vessels to more central vessels, contributing to a greater clearance of blood metabolites during hemodialysis^24^.

Our findings demonstrated that a combination of aerobic exercise and strength training can increase exercise tolerance time, since the meta-analysis included four studies that evaluated the duration of cardiopulmonary exercise testing and found significant results. The studies that used aerobic training alone prevented a similar analysis due to lack of data. However, Reboredo *et al*. (2011), who used aerobic training alone, found a significant increase in constant work-rate test time, which is considered as important as increased VO_2peak_ itself^6^.

Groussard *et al*. (2015) reported that greater increases in VO_2peak_ are related to training duration, and more significant changes were found in studies with exercise protocols of at least six months^12^. The training duration of most studies included in the present meta-analysis was longer than six months, which corroborates this statement.

The positive effects of intradialytic exercise on VO_2peak_ were verified through the pulmonary ventilation values obtained during the maximum cardiopulmonary stress test. These results demonstrated the reduced ventilatory work of hemodialysis patients who exercised^25^.

Pulmonary ventilation increases linearly with VO_2peak_ and reflects the demand, in liters per minute, of the volume of air forced in and out of the lungs during the maximal cardiopulmonary stress test. Ventilatory demand is influenced by an individual’s degree of physical deconditioning^9^.

Intradialytic exercise protocols are prescribed to improve the physical fitness of hemodialysis patients. Increased pulmonary ventilation is associated with cardiac performance, which influences the strength of the muscles involved in this mechanism, improving the respiratory system’s efficiency and, consequently, contributing to improved cardiopulmonary fitness^25^.

As a study limitation, we point out the low or very low quality of evidence of the included RCTs according to the GRADE evaluation.

## Conclusions

We can conclude that intradialytic exercise protocols can improve cardiopulmonary function, exercise tolerance and ventilatory efficiency in CKD patients, although a combination of aerobic and resistance training offers greater benefits.

## Supplementary information

Supplementary information

## Data Availability

All data generated or analyzed during this review are included in this published article (and its Supplementary Information files). The authors authorize the use of data for Scientific Reports.
