# Supplementary material for: Effects of intradialytic exercise on cardiopulmonary capacity in chronic kidney disease: systematic review and meta-analysis of randomized clinical trials
Source: Sci Rep. 2019 Dec 5;9:18470. doi: 10.1038/s41598-019-54953-x (PMC6895108; doi:10.1038/s41598-019-54953-x)
Supplement: Supplementary file 1 — Supplementary information [file 41598_2019_54953_MOESM1_ESM.pdf]

Effects of intradialytic exercise on cardiopulmonary capacity in chronic kidney disease: systematic review and meta-analysis of randomized clinical trials

Authors: Francini Porcher Andrade[1+*]; Patrícia de Souza Rezende[2+]; Tatiane de Souza Ferreira[1]; Gabrielle Costa Borba[1], Alice Mânica Müller[1+]; Paula Maria Eidt Rovedder[1,2,3+*]

1. Programa de Pós Graduação em Ciências Pneumológicas at Universidade Federal do Rio Grande do Sul (UFRGS), Porto Alegre, postcode 90040-060, Brazil.

2. Physiotherapy Course, Universidade Federal do Rio Grande do Sul (UFRGS), Porto Alegre, postcode 90040-060, Brazil.

3. Hospital de Clínicas de Porto Alegre, postcode 90035-903, Brazil.

* corresponding authors:
Francini Porcher Andrade (fran_porcher@hotmail.com) and Paula Maria Eidt Rovedder (larove_@hotmail.com).
+ these authors contributed equally to this work

Supplementary Information

Search strategy

For this study, terms sensitive for search ECR were used. The strategies used for each base descripted above.

Research strategy used in PubMed

#1	Renal Insufficiency, Chronic" OR "chronic Renal Insufficiencies" OR "Renal Insufficiencies, Chronic" OR "Chronic Renal Insufficiency" OR "Kidney Insufficiency, Chronic" OR "Chronic Kidney Insufficiency" OR "Chronic Kidney Insufficiencies" OR "Kidney Insufficiencies, Chronic" OR "Chronic Kidney Diseases" OR "Chronic Kidney Disease" OR "Disease, Chronic Kidney" OR "Diseases, Chronic Kidney" OR "Kidney Disease, Chronic" OR "Kidney Diseases, Chronic" OR "Chronic Renal Diseases" OR "Chronic Renal Disease" OR "Disease, Chronic Renal" OR "Diseases, Chronic Renal" OR "Renal Disease, Chronic" OR "Renal Diseases, Chronic"
#2	"Exercises" OR "Physical Activity" OR "Activities, Physical" OR "Activity Physical" OR "Physical Activities" OR "Exercise, Physical" OR "Exercises,

	Physical" OR "Physical Exercise" OR "Physical Exercises" OR "Acute Exercise" OR "Acute Exercises" OR "Exercise, Acute" OR "Exercises, Acute" OR "Exercise, Isometric" OR "Exercises, Isometric" OR "Isometric Exercises" OR "Isometric Exercise" OR "Exercise, Aerobic" OR "Aerobic Exercise" OR "Aerobic Exercises" OR "Exercises, Aerobic" OR "Exercise Training" OR "Exercise Trainings" OR "Training, Exercise" OR "Trainings, Exercise"
#3	"Exercise Tests" OR "Test, Exercise" OR "Tests, Exercise" OR "Cardiopulmonary Exercise Tests" OR "Arm Ergometry Test" OR "Arm Ergometry Tests" OR "Ergometry Test, Arm" OR "Ergometry Tests, Arm" OR "Test, Arm Ergometry" OR "Tests, Arm Ergometry" OR "Bicycle Ergometry Test" OR "Bicycle Ergometry Tests" OR "Ergometry Test, Bicycle" OR "Ergometry Tests, Bicycle" OR "Test, Bicycle Ergometry" OR "Tests, Bicycle Ergometry" OR "Fitness Testing" OR "Fitness Testings" OR "Testing, Fitness" OR "Testings, Fitness" OR "Step Test" OR "Step Tests" OR "Test, Step" OR "Tests, Step" OR "Stress Test" OR "Stress Tests" OR "Test, Stress" OR "Tests, Stress" OR "Treadmill Test" OR "Test, Treadmill" OR "Tests, Treadmill" OR "Treadmill Tests" OR "Physical Fitness Testing" OR "Fitness Testing, Physical" OR "Fitness Testings, Physical" OR "Physical Fitness Testings" OR "Testing, Physical Fitness" OR "Testings, Physical Fitness" OR "Cardiopulmonary Exercise Test" OR "Cardiopulmonary Exercise Tests" OR "Exercise Test, Cardiopulmonary" OR "Exercise Tests, Cardiopulmonary" OR "Test, Cardiopulmonary Exercise" OR "Tests, Cardiopulmonary Exercise"
#4	#1 AND #2 AND #3

Research strategy used in Cochrane

"Renal Insufficiency, Chronic" AND "Exercise" AND "Exercise Test"

Research strategy used in EMBASE

chronic kidney failure AND exercise AND exercise test [randomized controlled trial]

Table 1 - Risk of bias included studies

	Adequate sequence generation	Allocation concealment	Blinding of patients	Blinding of investigators	Blinding of outcome assessors	Description of losses and exclusions
Groussard et al, 2015	Yes	Unclear	No	No	No	Yes
Reboredo et al., 2011	Yes	Unclear	Unclear	Unclear	Unclear	Yes
Kouidi et al., 2010	Yes	Unclear	Unclear	Unclear	Unclear	Yes
Kouidi et al., 2009	Yes	Yes	Unclear	Unclear	Yes	Yes
Ouzouni et al., 2009	Yes	Unclear	Unclear	Unclear	Unclear	Yes
Petraki et al., 2008	Yes	Unclear	No	Unclear	Unclear	Yes
Konstantinidou et al., 2002	Yes	Unclear	No	Unclear	Unclear	Yes

Table 2 - GRADE

Certainty assessment							№ of patients		Effect		Certainty	Importance
**№ of studies**	**Study design**	**Risk of bias**	**Inconsistency**	**Indirectness**	**Imprecision**	**Other considerations**	**Exercise**	**control**	**Relative (95% CI)**	**Absolute (95% CI)**		
**$VO_{2\ peak}$ mL/Kg/min**												
7 [a,b]	randomised trials	not serious	very serious	not serious	very serious	none	125	118	MD **4.06 ml/Kg/L higher** (0.81 higher to 7.31 higher)		⨁◯◯◯ VERY LOW	
**Duration at the cardiopulmonary test**												
4	randomised trials	not serious	serious	not serious	serious	none	81	76	MD **3.1 min higher** (1.7 higher to 4.51 higher)		⨁⨁◯◯ LOW	
**Pulmonary Ventilation**												
4	randomised trials	not serious	serious	not serious	very serious	none	148	148	MD **13.1 liters higher** (7.12 higher to 19.09 higher)		⨁◯◯◯ VERY LOW	

CI: Confidence interval; **MD:** Mean difference
